# Gamification and Adherence to Web-Based Mental Health Interventions: A Systematic Review

**DOI:** 10.2196/mental.5710

**Published:** 2016-08-24

**Authors:** Menna Brown, Noelle O'Neill, Hugo van Woerden, Parisa Eslambolchilar, Matt Jones, Ann John

**Affiliations:** ^1^ Swansea University, Medical School Swansea United Kingdom; ^2^ Directorate of Public Health and Policy NHS Highland Inverness United Kingdom; ^3^ Centre for Health Science University of the Highlands and Islands Inverness United Kingdom; ^4^ Swansea University Department of Computer Science Swansea United Kingdom

**Keywords:** adherence, Web-based mental health interventions, well-being, gamification, engagement, dropout, patient compliance, patient nonadherence

## Abstract

**Background:**

Adherence to effective Web-based interventions for common mental disorders (CMDs) and well-being remains a critical issue, with clear potential to increase effectiveness. Continued identification and examination of “active” technological components within Web-based interventions has been called for. Gamification is the use of game design elements and features in nongame contexts. Health and lifestyle interventions have implemented a variety of game features in their design in an effort to encourage engagement and increase program adherence. The potential influence of gamification on program adherence has not been examined in the context of Web-based interventions designed to manage CMDs and well-being.

**Objective:**

This study seeks to review the literature to examine whether gaming features predict or influence reported rates of program adherence in Web-based interventions designed to manage CMDs and well-being.

**Methods:**

A systematic review was conducted of peer-reviewed randomized controlled trials (RCTs) designed to manage CMDs or well-being and incorporated gamification features. Seven electronic databases were searched.

**Results:**

A total of 61 RCTs met the inclusion criteria and 47 different intervention programs were identified. The majority were designed to manage depression using cognitive behavioral therapy. Eight of 10 popular gamification features reviewed were in use. The majority of studies utilized only one gamification feature (n=58) with a maximum of three features. The most commonly used feature was story/theme. Levels and game leaders were not used in this context. No studies explicitly examined the role of gamification features on program adherence. Usage data were not commonly reported. Interventions intended to be 10 weeks in duration had higher mean adherence than those intended to be 6 or 8 weeks in duration.

**Conclusions:**

Gamification features have been incorporated into the design of interventions designed to treat CMD and well-being. Further research is needed to improve understanding of gamification features on adherence and engagement in order to inform the design of future Web-based health interventions in which adherence to treatment is of concern. Conclusions were limited by varied reporting of adherence and usage data.

## Introduction

Common mental health disorders and poor well-being have significant economic, social, and individual costs [1-3]. The issue of promoting well-being while improving and managing mental health conditions remains a worldwide priority [4].

Web-based apps have been widely accepted and recognized as a cost-effective means by which to deliver proven and effective evidence-based therapies that were traditionally face-to-face, such as cognitive behavioral therapy (CBT), to improve mental health and well-being outcomes [5-9]. Web-based interventions provide an advantage over traditional face-to-face delivery due to their potential to reach wider populations through the removal of many access barriers, such as limited numbers of trained and available therapists, long waiting lists, high delivery costs, transportation, geographical issues, and social stigma attached to treatments [10-12].

An increasing number of Web-based platforms have been developed that provide treatment and resources for a wide range of conditions, common mental disorders (CMDs), serious mental health disorders, well-being, and lifestyle improvement. However, dropout and nonadherence are often high and vary widely. Reported rates of attrition range between 35% and 99% [13-18]. Context effects and health conditions influence adherence [19,20]. This is of critical importance because greater adherence to Web-based interventions is associated with improved mental health outcomes [21,22], whereas low adherence is reported to limit effectiveness of treatments [23].

A growing body of research has identified a range of technology-driven features that contribute to program adherence, quality, design, and usability of Web-based interventions [24,25]: persuasive technology [26], including “push factors” and short message service (SMS) text message notifications, alerts or personalized reminders [27,28], weekly tracking [29], incentives [30,31], interactive features [32], and social networks [33]. However, variation in reporting and measuring adherence has complicated understanding of the role of technological features [21].

Findings from the gaming literature have suggested that the inclusion and use of gamified features in Web-based health interventions may increase interest and enjoyment, improving user experience. This, in turn, may positively influence engagement and program adherence and encourage desired health behavior changes [34-38]. *Gamification* has been defined as “the use of game design elements in nongame contexts” [39]. It differs from *serious games*, which refers to the use of games in their entirety within nongaming contexts (as opposed to selected elements or individual features of a game). Thus, gamification is the use of individual features of game design applied in a context not usually associated with video gaming or game play. However, agreement of conceptual understanding remains debated [40] and academic opinion is varied. Gamification has enjoyed a recent explosion of success and increasing interest in a wide array of contexts beyond entertainment, health, education, news, and sustainability [41-43]. However, interest in game design has been researched in the fields of human-computer interaction and motivational psychology for much longer.

Recent research has called for the continued identification of features and “active” components that are most effective in improving program adherence while ensuring treatment remains effective [8,34,43]. A number of important adherence review studies have been published. For example, Kelders et al [19] identified predictors of high adherence such as randomized controlled trial (RCT) study design, frequency of counselor interaction (frequency of peer interaction was not found to predict adherence), more frequent updates and reminders, more extensive use of dialog support, and more frequent intended usage. In addition, van Ballegooijen et al [44] reported adherence to guided Internet CBT (iCBT) interventions for depression were equal to that of face-to-face delivery. Before that, Brouwer et al [45] reported that elements of interventions associated with human support (guided) were associated with higher adherence in physical health interventions. Schubart et al [46] identified that tailored advice, feedback, and guided programs increased user engagement in chronic health interventions. Earlier reviews focused on reporting the extent of the problem in the context of mental health interventions [18,47]. However, no prior reviews were identified that explicitly examined the role of gamification on adherence in the context of Web-based health interventions designed to treat CMD and improve well-being.

This review seeks to (1) explore, through systematic review of published peer-reviewed studies, the role of gaming features in Web-based interventions for the treatment of common mental health disorders or well-being and (2) to identify the “active ingredients” that influence treatment adherence.

### Objectives

The specific objectives of this study were to:

1. Identify studies that have incorporated gaming features into the design of their intervention to improve outcomes for CMDs and well-being;

2. Identify gamification features that influence adherence;

3. Report current rates of adherence;

4. Determine whether effects of the gamification feature on adherence varies across subgroup populations; and

5. Identify all terms commonly used to report adherence and maintenance with Web-based CMD and well-being and report the extent to which these are commonly reported in studies.

## Methods

### Protocol

This review was registered with PROSPERO on April 16, 2015 (CRD42015017689).

### Procedure

A comprehensive search of seven electronic databases was conducted: Medline (Ovid interface), PsychINFO (Ovid interface), Cochrane Library, the Cumulative Index to Nursing and Allied Health Literature (CINHAL; EBESCO interface), Business Source Complete (EBSCO interface), Inspec (Ovid interface), and the ACM Digital Library. Search dates were between database inception and April 2015. Search strategies were customized for each database.

A combination of search terms were used to identify all relevant articles under the following categories: “Web-based,” “intervention,” “CMD/well-being,” and “adherence” (Multimedia Appendix 1).

### Inclusion Criteria

The inclusion criteria included:

1. The study must have included one or more gamification feature in the intervention;

2. The study was designed to manage any CMD or improve well-being (including physical conditions that report CMD/well-being outcome);

3. The intervention was delivered via the Web (Internet);

4. The intervention was designed to be accessed on more than one occasion;

5. RCT study design; and

6. The study must have reported at least one measure of attrition, adherence, engagement, dropout, or other term referring to such.

### Exclusion Criteria

The exclusion criteria were (1) the intervention was delivered via paper, face-to-face, CD-ROM, or other non-Web-based method and (2) participants were younger than age 18 years.

### Gamification

The definition of *gamification* used in this review was “the use of game design elements in nongame contexts” [39]. Ten gamification features were reviewed. The features reviewed were those identified by Cugelman [36]. These were informed by Hamari et al [48] and are described in Multimedia Appendix 2. Two authors (MB, AJ) discussed the selection of this list.

### Review Process

Two reviewers (MB, NoN) independently reviewed the title for relevance, then the abstract against inclusion/exclusion criteria. A third reviewer (HvW) resolved any disagreements. Measures of agreement were calculated (kappa statistic). Full-text articles of those included were retrieved at this stage. Two reviewers (MB, NoN) independently reviewed each article. Each was assessed against the inclusion/exclusion criteria outlined previously. The first instance where it did not meet eligibility was recorded as the reason for exclusion and the study was not assessed against additional inclusion criteria [49]. Reviewers discussed all articles that were not unanimous (see the PRISMA flowchart in Multimedia Appendix 3).

### Data Extraction

A data extraction form was developed and piloted with five studies meeting the inclusion criteria. The following data were extracted for review (MB):

1. Participant characteristics: including recruitment setting, use of diagnostic interview, total number of participants randomized to intervention, sample size, gender, and age.

2. Intervention characteristics: including intervention name, number of trial arms, primary condition, therapeutic approach, intended duration (weeks), modules to be completed, automated or guided delivery, format of delivery, and outcome measures used.

3. Interactive elements of intervention: including automated email reminders, interactive quizzes, social networking (community forum), homework, or diary tasks.

4. Gamification features: a record of the feature(s) used in the intervention design.

5. Adherence: including adherence to study protocol, completion rate, and term used to refer to adherence.

### Assessment of Risk of Bias in Included Studies

The quality of each included study from a risk of bias perspective was assessed (NoN) using the Cochrane Collaboration Risk of Bias tool as described in the *Cochrane Handbook for Systematic Reviews of Interventions* [50]. Each included study was assessed against the six bias domains and source of bias subdomains outlined in order to produce a summary risk of bias assessment score (low, high, or unclear). The majority risk level in each subdomain was utilized and summarized across all domains. If there were four or more subdomains with a low risk of bias, then it would be judged that the study showed an overall low risk of bias.

### Data Analysis

Descriptive and exploratory analyses were conducted in SPSS 22 (IBM Corp, Armonk, NY, USA) and Review Manager 5.3 (RevMan 5.3; The Cochrane Collaboration, Copenhagen, Denmark).

An adherence rate to study protocol was calculated as the principal summary measure. A percentage score for adherence to each intervention was calculated to allow comparison across interventions. This was the percentage of those completing postassessment by the number of participants initially randomized (to an intervention trial arm) because limited data were available on total completion rate of interventions.

A series of procedures were carried out. First, the adherence rates of interventions using only one gamification feature were visually presented in a series of forest plots, shown in comparison to adherence rates for inactive controls (where available). The mean adherence rate of interventions using only one gamification feature was calculated by adding the adherence rate for each study that used this feature and dividing it by the number of these studies. A one-way ANOVA was conducted to identify statistical differences between adherence rates for studies using different, single gamification features. Second, adherence rates for interventions using one, two, or three (total number of) gamification features were similarly calculated and presented visually in a bar chart. Forest plots showing adherence compared to inactive control (where available) are also presented. A one-way ANOVA explored statistical differences in adherence. Third, the mean adherence rate was calculated per condition and displayed in a bar chart. A one-way ANOVA explored statistical differences in adherence per condition. Finally, following these comparisons, an independent *t* test was conducted to examine statistical differences in adherence as a result of additional interactive features (in dichotomous features; ie, sequential or free navigation and automated or guided delivery). A one-way ANOVA was conducted to explore differences in features which included three or more categories (intended duration and modules, total number of interactive intervention characteristics). Values within each were recategorized to form three distinct categories.

A standard multiple regression analysis was performed to explore the role of interactive intervention characteristics in explaining adherence. Independent variables were entered into the model as a block using the enter method (total number of gamification features, guided or automated, sequential or free navigation, intended duration, modules, and total number of interactive intervention features). Adherence was entered as the dependent variable. It is recommended that 15 cases be included per predictor variable in social sciences [49].

## Results

### Summary Data

After duplicates were removed, 2170 titles and 774 abstracts were reviewed. Following full-text review, 61 RCTs remained (Multimedia Appendix 3). The kappa statistic showed good agreement between reviewers at the title and abstract stage (κ=.933 and κ=.694, respectively).

In all, 47 RCTs were two-armed trials, 12 were three-armed trials, one was a four-armed trial, and one was a six-armed trial. Of the two-armed trials, 21 compared to a wait-list control group, three to treatment as usual, one to placebo, one reported no treatment, and 20 used an active comparator. These included 11 interventions and nine attention controls. Of the 12 three-armed trials, two compared to two inactive controls, nine compared to an active intervention plus wait-list control or treatment as usual, and one included two interventions using different therapeutic approaches. The four-armed trial compared to a Web-based intervention plus tracking and two inactive conditions. The six-armed trial consisted of six active interventions. Multimedia Appendix 4 provides a full reference list of all 61 included articles and a summary of intervention characteristics of all 82 included arms (where no arm is recorded this is to indicate it was the additional trial arm in an RCT).

### Cochrane Risk of Bias Score

**Figure 1:**
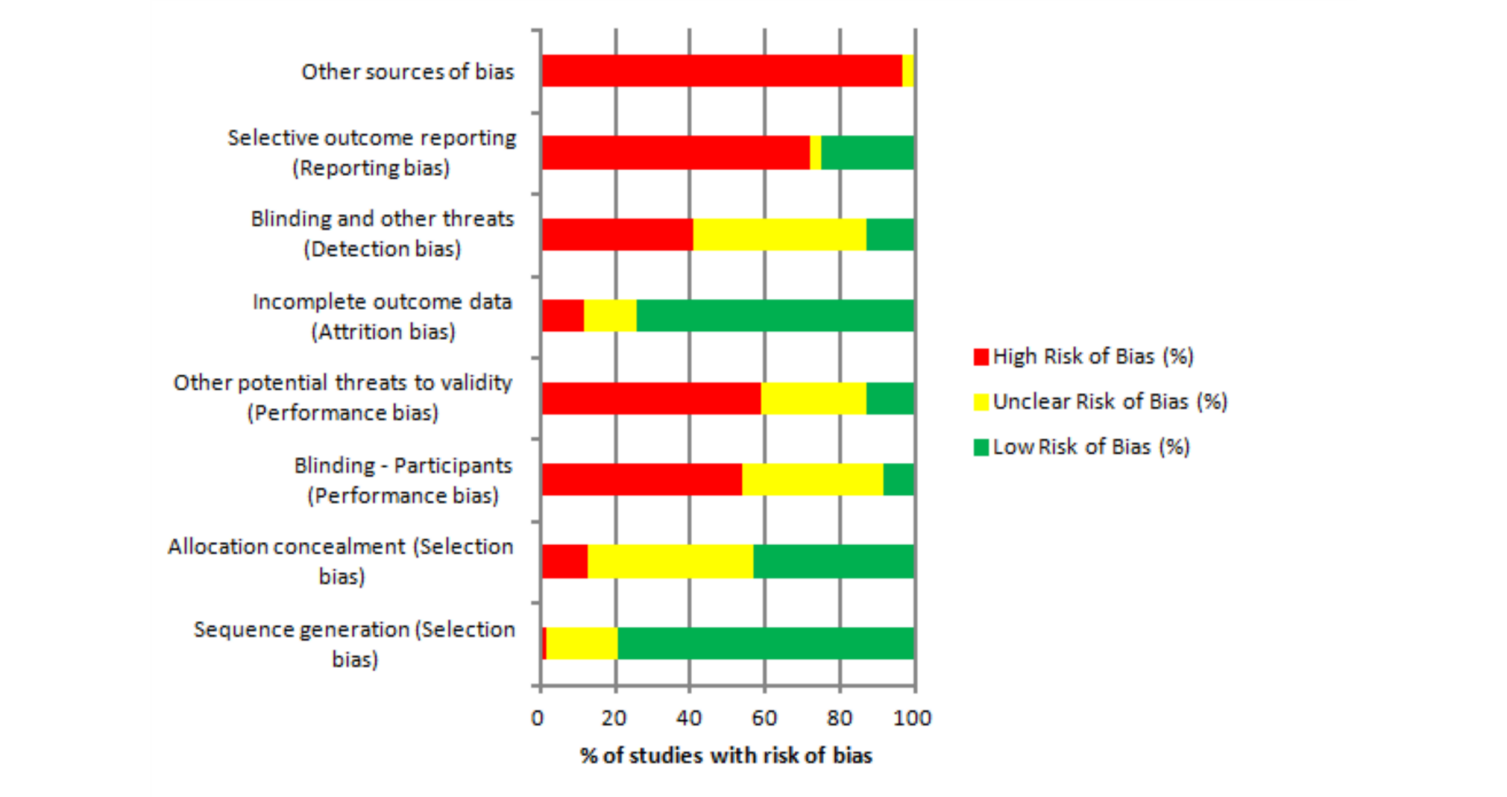
Risk of bias assessment of included articles (N=61).

Of the 61 RCTs included in this systematic review, 37 (61%) were judged to be of high risk of bias, eight (13%) were judged to be of low risk of bias, and an unclear risk of bias was assigned to 16 (26%) of the included studies (Figure 1). The quality of the evidence provided within the included studies was variable. Sources of bias included inconsistent implementation of interventions, follow-up methods, completion rates, and studies being underpowered to statistically detect intervention effects, and self-selected study populations (Figure 1).

### Descriptive Statistics

The main results table presented in Multimedia Appendix 4 reports a summary of key characteristics for all included intervention arms (n=82).

#### Participant Characteristics

Across the 61 RCTs, 14,726 participants were randomized to either an intervention or control condition. The RCTs varied widely in size from a total of 24 to 23,213 randomized participants. Overall, 41 RCTs had sample sizes less than 200, 15 had sample sizes between 200 and 999, and five had sample sizes more than 1000. Four RCTs included females only. Four RCTs restricted inclusion to those older than 45 years and one included a sample of those between the ages of 18 and 24 years; the remainder (n=56) recruited from age 18 years and older. Participants were recruited from the general population (n=39), clinical populations (n=11), students (n=4), military (n=2), and organizational workplaces (n=5). The majority of RCTs were conducted in Australia (n=20) and the United States (n=18). The majority of participants self-referred into a trial (87%).

#### Intervention Arms

From the 61 RCTs, a total of 82 active intervention arms were identified. As such, the following section presents the adherence and gamification results from 82 interventions.

#### Condition

Interventions were designed to treat a range of symptomology: depression (n=30), depression with comorbid anxiety (n=5), anxiety including social anxiety disorder and generalized anxiety disorder (n=9), well-being (n=7), social phobia (n=7), posttraumatic stress disorder (n=4), obsessive compulsive disorder (n=1), panic disorder (n=1), stress (n=3), binge eating disorder (n=1), and physical conditions (n=14). A total of 37 interventions reported use of clinical diagnostic interview.

The 14 interventions designed to manage physical conditions were physical activity (n=3), smoking cessation (n=1), sexual dysfunction in female cancer patients (2), headache (n=2), insomnia (n=4), and weight loss (n=2). Pre- and postoutcome measures for a CMD or well-being were reported in each of these trials.

#### Intervention Characteristics

All interventions were Web-based and available via personal computers, laptops, and Internet-enabled devices. In total, 47 different therapeutic interventions were identified and a number of these were utilized in successive RCTs: MoodGYM (n=6), Beating the Blues (n=3), MoodGYM and BluePages combined (n=2), deprexis (n=2), SHUTi (n=2), and The Shyness Program (n=5). In this review, “intervention” refers to the Web-delivered therapeutic treatment program.

##### Automated/Guided

Of the 82 interventions, 50 were automated. Automated delivery of an intervention refers to the use of an intervention treatment program without any human support. The remainder (n=32) were guided. Guided delivery refers to support of a human guide during the course of the treatment. Guided interventions included a range of guided interactions: therapeutic telephone contact (n=13), face-to-face therapy (n=5), and therapeutic emails (n=21).

##### Therapeutic Approach

In total, 59 interventions were based primarily on CBT, one of which used CBT in combination with psychoeducation and interpersonal psychotherapy, two used cognitive restructuring without behavioral activation, two used mindfulness, two used positive psychology, one was based on a stress and coping model, two used Internet psychotherapy, five employed health behavior change techniques, and nine did not specify a therapeutic approach. Some studies noted additional elements used in the intervention. These included cognitive bias modification online (n=1), Internet-delivered supportive counseling (n=1), psychoeducation (n=2), interpersonal therapy (n=2), problem solving (n=2), motivational interviewing or motivational principles (n=2), and physical activity (n=1).

##### Format of Delivery

In all, 63 intervention arms were released sequentially in a predetermined order over time, 16 could be freely navigated, two [51,52] presented modules in sequence but allowed participants free navigation, and one [53] included free navigation once a specific module had been completed.

##### Duration

The duration of the interventions ranged between 3 and 20 weeks (mean 7.8, SD 2.4). One did not specify the intended duration [54], although it clearly stated that the intervention was to be used more than once. Many were eight (n=25), six (n=22), or 10 (n=8) weeks in duration.

##### Modules

The number of modules within each intervention ranged between zero and 13 (mean 6.4, SD 2.6). Three did not use a modular format. Most interventions included six (n=30), eight (n=11), or five (n=9) modules.

##### Interactive Intervention Elements

Information available regarding interactive elements employed in each intervention varied. Text was presented in all, accompanied by a range of additional elements, automated email reminders (n=36), SMS text message reminders (n=13), telephone reminders (n=12), interactive quizzes (n=37), social media (n=11), and homework (n=47).

#### Gamification

Eight of 10 gamification features reviewed were identified in use: story/theme, progress, feedback, goal setting, rewards, challenge, badges/trophies, and points. No study incorporated levels or game leaders. The majority of interventions used only one gamification feature (n=58); the maximum number used in any one intervention was three. Of the interventions employing only one gamification feature, story/theme was most commonly used (n=33), followed by progress (n=10), goal setting (n=6), rewards (n=6), and feedback (n=3). Of those using more than one feature (n=24), 19 used two features and five incorporated three features.

#### Adherence

A wide variety of terms were used to report a measure of adherence: adherence, attrition, dropout, noncompleters, lost to follow-up, participant withdrawal, nonresponse, completion rate, did not complete, retention rate, loss, and compliance.

Overall adherence to study protocol ranged between 3.37% and 100% (n=82, mean 71.7%, SD 20.3%). Adherence to control groups ranged from 5.98% to 100% (n=58, mean 78.2%, SD 19.1%). The mean adherence rate of studies excluded for not including a gamification feature was 75.2% (SD 19.6%) with a range of 5.3% to 100%. There were differences between the ways in which studies classified adherence and reported their data, making meaningful comparison complicated. The limitations of such are addressed in the Discussion.

Reasons for nonadherence were provided in 33 RCTs. The following reasons were provided: lack of time, disinterest, no need for treatment, hardware or technical issues, program perceived as noneffective, life events, felt better after a few modules, disappointed by group assignment, holiday, work commitments, poor health, and no longer wish to participate. One RCT [55] reported removal of 19 participants due to fraudulent participation. One RCT only reported data for those participants who completed the entire intervention (due to a programming error).

#### Usage Data

Limited usage data were reported, mean number of modules completed (n=39 reported this data), program completion (n=45), with a mean completion rate of 54.0% (SD 24.6%), and log data. The way in which log data was reported varied further; mean time spent per visit in minutes (n=4), mean log-on rate (n=5), total time duration (n=2), total page views (n=1), and activities opened (n=1).

### Statistical Analysis of Intervention Characteristics and Adherence

#### Gamification

Adherence was examined per gamification feature for those interventions that employed only one gamification feature (n=58). Forest plots present the adherence per intervention arm in comparison to its control condition (where a control condition was used as opposed to an active intervention). The following forest plots show two columns: the intervention arm and the control group. The term “events” refers to the number of randomized participants remaining at postassessment, whereas “total” refers to the total number randomized to that intervention at the start of the trial. If a score of zero is recorded, either the data was unavailable or there was no control group to compare against. For example, in some RCTs the comparator group was another (treatment) intervention or a modified version of the same intervention. The weight is automatically calculated by RevMan based on the total number of participants in the trial. A mean adherence is also reported; this does not include the control arm data (unlike the forest plots).

#### Goal Setting

**Figure 2:**
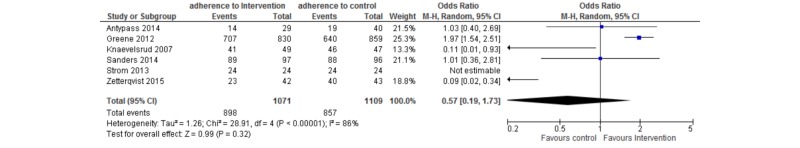
Forest plot showing the adherence rate of interventions using goal setting as a gamification feature.

Goal setting was defined as users informed of a goal or are required to establish their own goals to achieve over the duration of the program (intervention). Six interventions incorporated goal-setting activities. Adherence compared to control is shown in Figure 2. Mean adherence for the six interventions was 72.3% (SD 22.8%).

#### Progress

**Figure 3:**
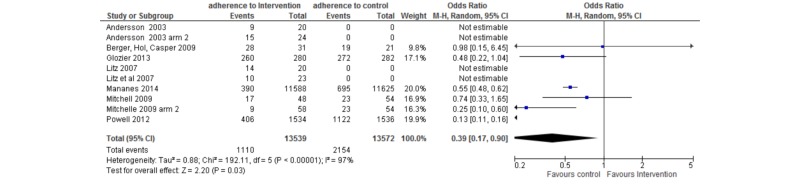
Forest plot showing the adherence rate of interventions using progress as a gamification feature.

Progress was defined as progression through the program or game. Participants could monitor progress with self or others. Ten interventions incorporated progress. Adherence compared to control is shown in Figure 3. Mean adherence was 53.5% (SD 31.2%).

#### Feedback

**Figure 4:**
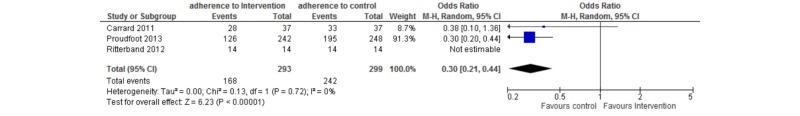
Forest plot showing adherence of interventions employing feedback as a gamification feature.

Feedback was defined as automated feedback provided on progress. Three interventions incorporated automated feedback. Adherence compared to control is shown in Figure 4. Mean adherence was 75.9% (SD 24.0%).

#### Rewards

**Figure 5:**
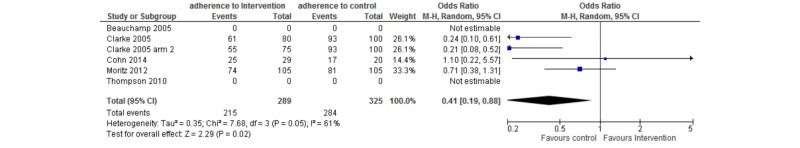
Forest plot showing adherence of interventions employing rewards as a gamification feature.

Rewards for achievement included in-game goods or artifacts (functional or nonfunctional to the program). Six interventions utilized rewards. Adherence compared to control is shown in Figure 5. Mean adherence was 72.1% (SD 13.3%).

**Figure 6:**
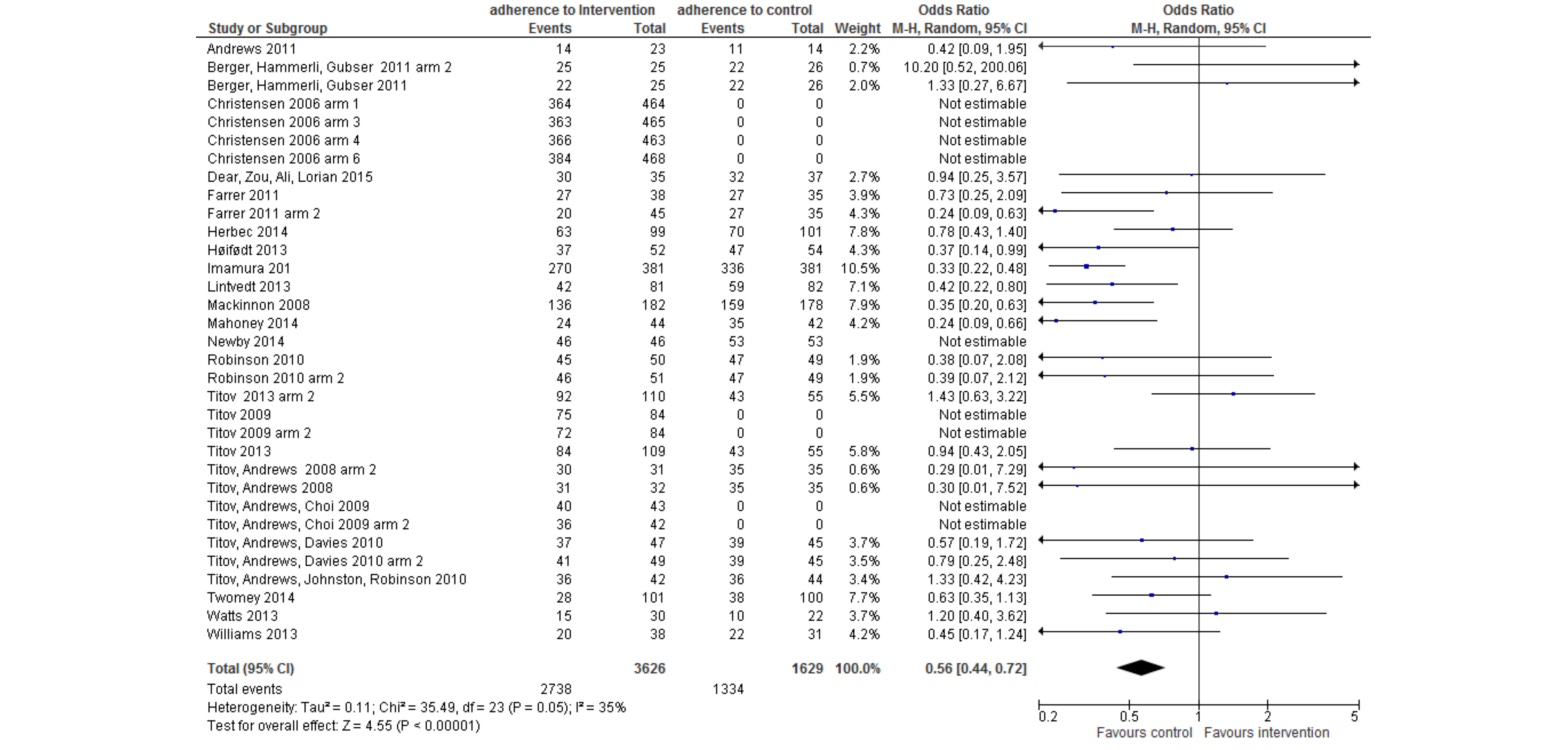
Forest plot showing adherence of interventions employing story/theme as a gamification feature.

#### Story/Theme

A story/theme included fun and playfulness, playing out an alternate reality, an avatar, or an illustrated story. In all, 33 interventions used a story/theme feature *.* Adherence compared to control is shown in Figure 6. Mean adherence was 76.3% (SD 17.0%).

A one-way ANOVA did not reveal any statistical differences between interventions using the preceding gamification features (n=58, *P*=.19).

#### Comparison of Adherence Rates per Use of Total Number of Gamification Features

The mean adherence rates for interventions incorporating one, two, and three gamification feature were 71.5% (SD 21.6%), 70.5% (SD 17.9%), and 78.2% (SD 12.3%), respectively. A one-way ANOVA did not reveal any statistically significant differences (*P*=.74). Adherence compared to control are displayed in three forest plots to visualize differences in studies employing one, two, and three gamification features (Multimedia Appendix 5).

#### Gamification Use by Condition

Multimedia Appendix 6 shows the frequency each individual gamification feature was employed in an intervention per condition. The total number is more than 82 because some interventions used two or more features. The mean adherence rate per condition is presented in Multimedia Appendix 7. One-way ANOVA did not reveal any statistical differences (*P*=.18).

#### Examination of Additional Intervention Characteristics

Delivery format, such as sequential (n=65, mean 72.1%, SD 21.3%) and free navigation (n=17, mean 70.2%, SD 16.2%), did not influence adherence to intervention (*P*=.20). Automated interventions had a mean adherence of 67.9% (n=50, SD 21.8%) compared to guided interventions (n=32, mean 77.5%, SD 16.2%). An independent *t* test did not reveal this to be statistically significant (*P*=.05).

One-way ANOVA did not reveal any statistical difference for intended duration (6 weeks: mean 65.1%, SD 24.3%; 8 weeks: mean 74.0%, SD 17.3%; 10 weeks: mean 76.4%, SD 17.1; *P*=.15), number of modules (<6 modules: mean 70.5%, SD 24.3%, 7-9 modules: mean 73.5%, SD 17.3%; ≥10 modules: mean 73.6%, SD 17.1%; *P*=.80), or total number of interactive features (0-2 features: mean 67.5%, SD 22.3%, 3-4 features: mean 77.5%, SD 15.3%; 5-6 features: mean 77.8%, SD 19.0%; *P*=.08).

Standard multiple regression indicated that the independent variables only explained 10.3% (*P*=.22) of the variance in adherence rate.

## Discussion

This review sought to identify RCTs that incorporated gaming features into the design of Web-based health interventions to treat CMDs or well-being. Physical health interventions that included an outcome measure for CMD or well-being were included when identified. This is the first review that has examined the use and role of gamification features on adherence in this context. Ten key gamification features were examined [36].

A total of 61 RCTs comprising 82 intervention arms were analyzed and 47 separate interventions were identified. Interventions designed to treat depression, which were intended to be 8 or 6 weeks in duration, incorporating six modules, and utilizing CBT were most common. This is shorter than the typical 10-week duration identified previously [19]. The most common format of delivery was a weekly sequential release of modules. Interventions allowing free navigation were less common. Interventions were more likely to be automated rather than guided. The majority of RCTs were found to have a high risk of bias.

One aim was to explore whether gamification features have been incorporated into the design of interventions developed to manage CMD or improve well-being. This review identified eight gaming features in use. The majority of studies used only one feature (goal setting, progress, feedback, reward, or story/theme). No studies specifically compared the impact of different gamification features on program adherence in the same RCT; however, one trial compared six versions of the same intervention (MoodGYM). Two of these trial arms were found to incorporate two gamification features, whereas the remaining four arms only included one [56]. However, the purpose of the trial was not to compare use of these features. Overall, the most common feature utilized was story/theme *.* Interventions using this did not commonly incorporate additional features; only six were found which did [56-61]. Progress and feedback were used together in six interventions [27,56,62-65]. Points and challenge were not frequently implemented and levels and game leaders were not incorporated at all.

The main aim of this review was to explore whether incorporating gamification features into the design of these interventions influenced adherence to treatment. In order to examine this, adherence was examined first. Adherence to intervention was lower overall than adherence to control when control was inactive (means 71.7% and 78.2%, respectively). Previous reviews reported higher adherence to guided interventions compared to automated interventions [45]. This review supported this (77.5% and 67.9%, respectively) lending further support for the role of guides in self-help treatments. However, this difference was not statistically significant.

Looking at the role of gamification features, adherence rates were compared across those using different features when only one feature was incorporated. No statistical difference was observed, which supported use of one single feature over another, despite the mean adherence rates ranging from 53.5% to 75.9% for progress and feedback, respectively. Nor was there any significant difference found between studies using different total numbers of gamification features (one, two, or three features). However, the forest plots suggest that as additional features are added, adherence moved closer to favoring the intervention over control.

An additional aim of this review was to determine whether adherence to interventions using gamification differed across health conditions. Interventions designed to treat social phobia had higher adherence than those designed to treat well-being (*P*=.048). However, no other statistical difference was observed. Findings reported here are in line with established published findings. Kelders et al [26] reviewed the impact of persuasive features and system design. They characterized typical studies and identified that RCT design, more frequent usage, updates, and dialog support predicted higher adherence. Interventions covered lifestyle, physical health, and mental health programs. Health care context did not predict adherence.

As a result, additional intervention features were also examined in an effort to shed light on active ingredients influencing adherence. Again no statistically significant differences were observed and none of the variables were found to explain any significant proportion of the variance in adherence rate (total variance explained was only 9.4%). However, mean adherence increased as intended duration increased from 6 or 8 weeks to 10 weeks’ duration.

Criticisms of gamification have been levied and discussed in the literature [66]. For example, a Gartner report [67] stated “gamification is currently driven by novelty and hype,” whereas Bogost [68] considered it a quick fix adopted by businesses to increase and promote engagement. Underpinning these criticisms is the concern that implementation of individual features such as points and leader boards actually miss the real essence and power of games as motivational techniques, which have the potential to positively encourage behavior change [69] or positively encourage adherence to treatment programs that reduce individual suffering through reductions in clinical symptoms. Although many studies were found to have incorporated one game feature into their treatment program, it is possible that such negative opinions may have reduced wider application in this health context due to concerns of appropriateness. However, Cugleman [36] highlighted that gamification, like other persuasive architectures, has merit if implemented in the right way.

It is important to consider the way in which gamification features identified in use were incorporated into intervention designs. There were only three examples in which the use of game mechanics was clearly acknowledged and the intention of use identified as a means to address and increase user engagement and enhance enjoyment. Cobb and Porier [70] used in-game rewards, badges, and challenges to engage participants in a daily challenge to improve well-being. In this example, adherence was high and usage data well reported. More than half the participants continued to engage with the program at 60 days and 92.4% were reported to have completed one challenge. Authors reported a positive dose-response relationship for well-being in which higher program engagement predicted better well-being at postassessment and follow-up. Similarly, a guided physical activity intervention that assessed well-being outcomes applied motivational principles and game elements, including visualization of progress and automated goal setting activities, specifically to enhance engagement and participation [55]. Imamura et al [51] incorporated comic strip stories in an effort to “foster learner’s interest in the program” (p 3). However, the remaining interventions did not commonly acknowledge or describe their use of gamification features. For example, Titov et al [61] implemented story/theme, goal setting, and challenge in The Shyness Program, without acknowledgement that game mechanics were incorporated in the intervention. Indeed incorporation of such features may not have been considered (by those who developed the intervention) to represent implementation of game mechanics. Further examples include Sheeber et al [71] who incorporated three features, without recognition of such, in a guided intervention to manage maternal depression. In this example, intervention design and development was focused on principles that promoted self-regulated learning. Adherence and completion rate was high (97% and 63%, respectively). Intervention descriptions focused on the theoretical basis rather than the technological aspects of development. The intentional use of game design elements has recently been suggested as a defining feature of the operationalization of gamification [40]; as such, this highlights the potential importance of intended use in operationalization of features. Doherty et al [32] outlined the importance of encouraging engagement with, or adherence to, treatment rather than technology and that it is important to bear this in mind during discussion on use of gamification features in this context in which the ultimate intention is to alleviate suffering and improve well-being.

### Strengths and Limitations

This review was based on an extensive search of a large number of health and computer science databases. Hand searching was not conducted, but the expertise of the multidisciplinary team means that although publication bias cannot be excluded, this comprehensive review did identify a large number of relevant studies.

This review aimed to explore the potential role of gamification to increase program adherence and engagement, adherence being an issue that has plagued Web-based health interventions for some time [47,72]. In order to examine the role of gamification on adherence, adherence to study protocol was used. This was considered an objective, comparable measure calculated as a percentage of those (randomized) who completed postassessment outcome measures. Although this is useful, it offers less insight than module completion rates would. However, limited reporting of data, such as log-on rates, module completion, and mean access time, meant this was not possible. Only 34 studies reported a percentage for program completion and only 10 provided data for log-on rates, with one exception [73]. These studies were all reported after 2009. A more comprehensive and standardized usage report across trials would assist and inform further analyses of adherence and program engagement. This finding is in line with previous discussion on adherence reporting [19,62,74]. Morrison and Doherty [74] provided a useful analysis of log data that could be replicated in future studies.

Interventions evaluated via RCT methodology was a specific inclusion criteria of this review; as such, it is possible that a body of literature pertaining to management of CMD or well-being that incorporate gamification features may have been excluded. However, RCTs follow robust methodological procedures and are considered to provide the highest quality evidence, so the approach adopted is of value [75].

Varied reporting complicated initial identification of studies for inclusion. Not all studies provided a detailed description of the intervention programs. However, seven provided clear, detailed descriptions of intervention features, including screenshots and illustrations [27,51,70,76-78].

Interventions using gamification features in conditions other than depression were small in number, which limited opportunity to explore the influence of gamification features on adherence across health conditions.

Furthermore, the way in which specific gamified features were incorporated warrants discussion. In this study, rewards were commonly seen to be financial in nature, whereas progress was often controlled progression through the system. Goal setting and feedback were aligned with established strategies used in therapeutic treatment of CMD and their role is well defined in terms of supporting and encouraging behavior change. In reviewing intervention designs, it was not always possible to identify the intention behind each feature and they are also commonly used features in Web-based programs. However, they were not employed in all interventions and so remain of interest in this context.

It is important to acknowledge that adherence also may be influenced by additional factors that could not be assessed in this review. This is highlighted in the small variance rate (10.3%). Furthermore, attrition to mental health treatments is also experienced in face-to-face delivery formats.

### Implications for Practice

Future research should look to examine whether application of specific gamification features influences adherence to protocol and completion rate. No RCT was identified that specifically considered the role of gamified features on promotion of adherence to mental health programs. This could be achieved through comparisons of the same intervention (in the same clinical population) adjusted to include either different gamification features, different combinations of gamification features, increasing numbers of gamification features, or use of one specific gamification feature compared to none. Studies looking to explicitly make these comparisons may shed further light on the role of individual features extracted from game design on adherence to Web-based health interventions. These effects should also be explored across different health and well-being contexts to identify whether inclusion of gamification features are more or less effective at increasing engagement and adherence across different patient populations and subgroups, such as different levels of clinical symptomology.

It would also be beneficial to explore the use of gamification in interventions based on alternative therapies to that of CBT (which comprised the majority of those reviewed here); for example, whether they have a role to play in encouraging engagement to interventions based on acceptance and commitment therapy. In addition to this, future research might benefit from exploration of gamification in interventions, allowing free navigation as opposed to a linear, weekly format as identified here. This may shed further light on the potential role of game mechanics on program engagement and adherence to treatment.

Assessment of participant’s motivation to complete the full intervention on entering the program might also offer an alternative way to explore the role of gamification. Use of extrinsic motivation features may influence some people more than others. Exploration of people’s reasons for participating at the onset of a RCT might shed light on the role of gamification features. Gamification promotes motivation through external means, which means those who are internally motivated may not be influenced to the same extent.

Research findings have indicated that higher adherence is associated with increased treatment effectiveness (dose-response relationship). Some have discussed a beneficial level of engagement that facilitates a positive health outcome [32] and this is certainly an area for future interest. This was not examined in this review, but could be further explored in relation to the inclusion of gamification features.

### Conclusion

Gaming features have explicitly been implemented into the design of interventions to treat CMDs and well-being. However, this was not common. This review did not find any evidence that use of specific gamification features was associated with higher adherence to the intervention program as measured by adherence to protocol. Furthermore, no evidence was found to suggest that interventions incorporating additional gamification features had any statistically significant influence on adherence. However, no studies explicitly examined the role of gamification on program adherence or engagement.

What the review did show was that guided interventions and interventions intended to last 10 weeks, as opposed to 6 or 8 weeks duration, and those incorporating three gamification features had a higher mean adherence rate. This may provide initial insight into the design of future interventions wishing to utilize gamification features in an attempt to address adherence and contribute to the ongoing discussions surrounding the use of game design elements in nongame contexts.

## Appendix

Multimedia Appendix 1 Keyword search table.

Multimedia Appendix 2 Gamification features.

Multimedia Appendix 3 PRISMA Flow diagram of included and excluded studies.

Multimedia Appendix 4 Main results table and references of included studies.

Multimedia Appendix 5 Forest plots showing mean adherence in studies using one, two and three gamification features.

Multimedia Appendix 6 Gamification features used per condition.

Multimedia Appendix 7 Mean adherence rate per condition.

## Abbreviations

CBT: cognitive behavioral therapy
CMDs: common mental disorders
iCBT: Internet cognitive behavioral therapy
RCT: randomized controlled trial
SMS: short message service
