# Supplementary figures and images for: Gamification and Adherence to Web-Based Mental Health Interventions: A Systematic Review
Source: JMIR Ment Health. 2016 Aug 24;3(3):e39. doi: 10.2196/mental.5710 (PMC5014987; doi:10.2196/mental.5710)

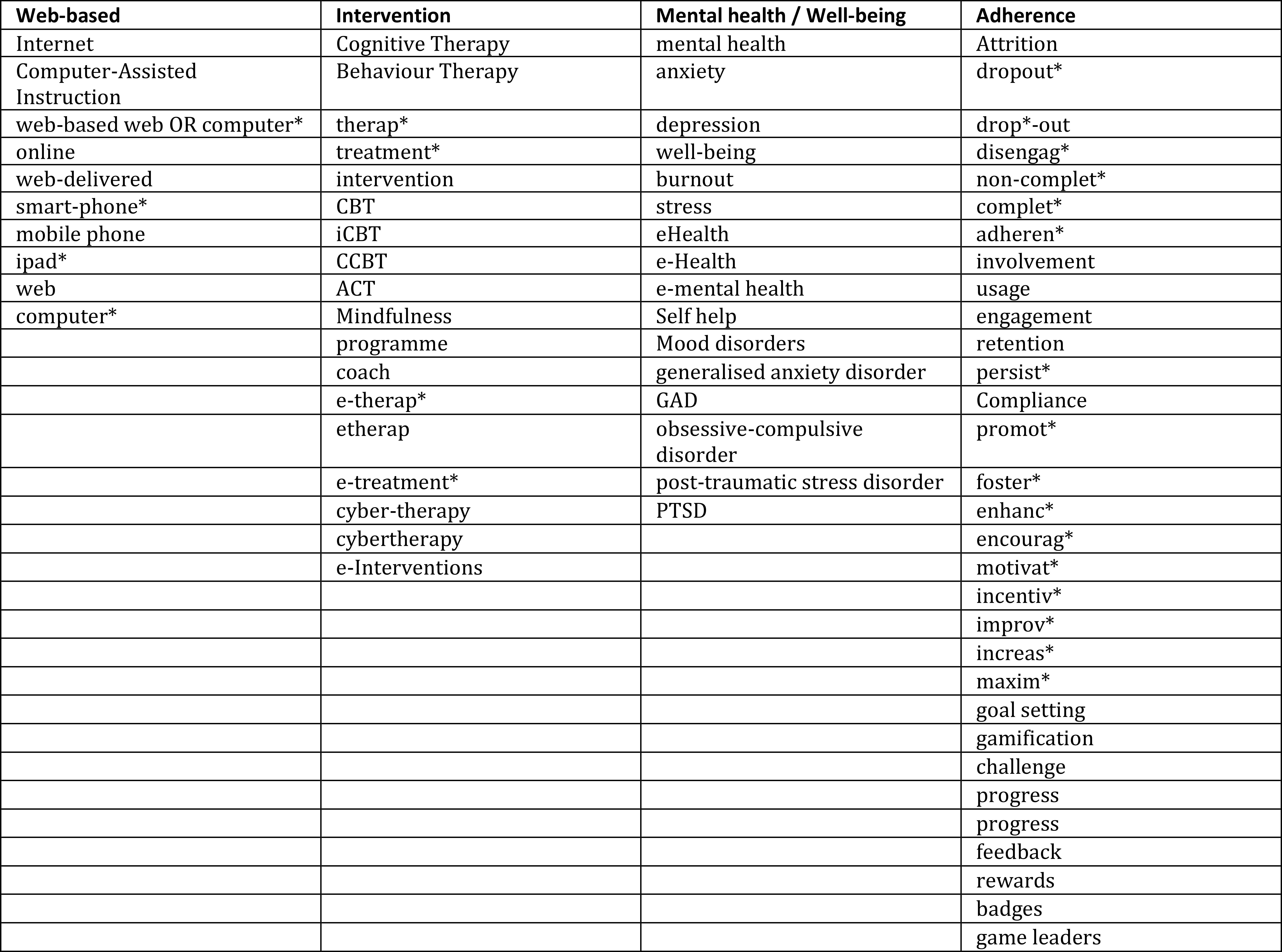

Supplement: Multimedia Appendix 1 [file mental_v3i3e39_app1.png]

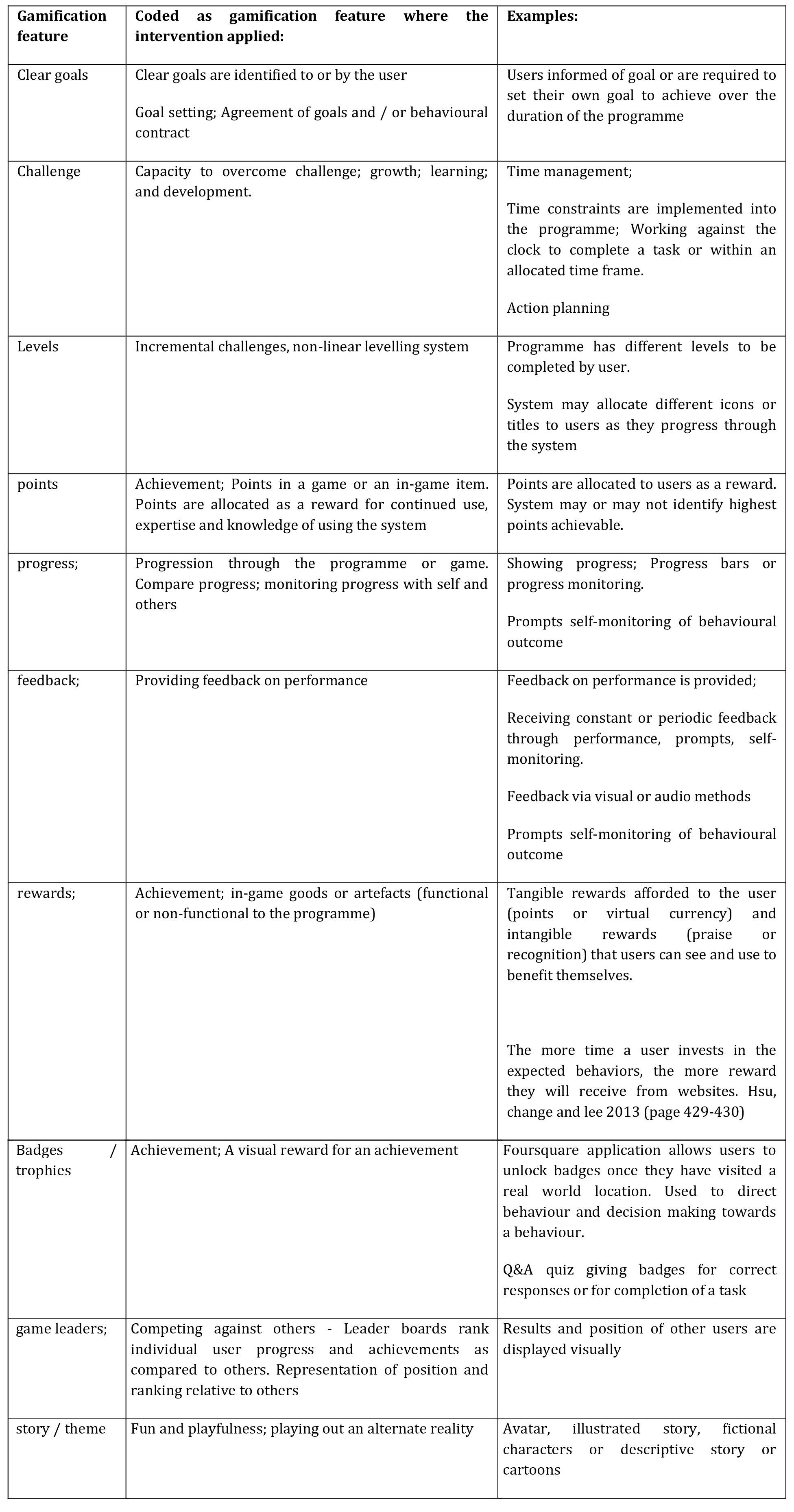

Supplement: Multimedia Appendix 2 [file mental_v3i3e39_app2.png]

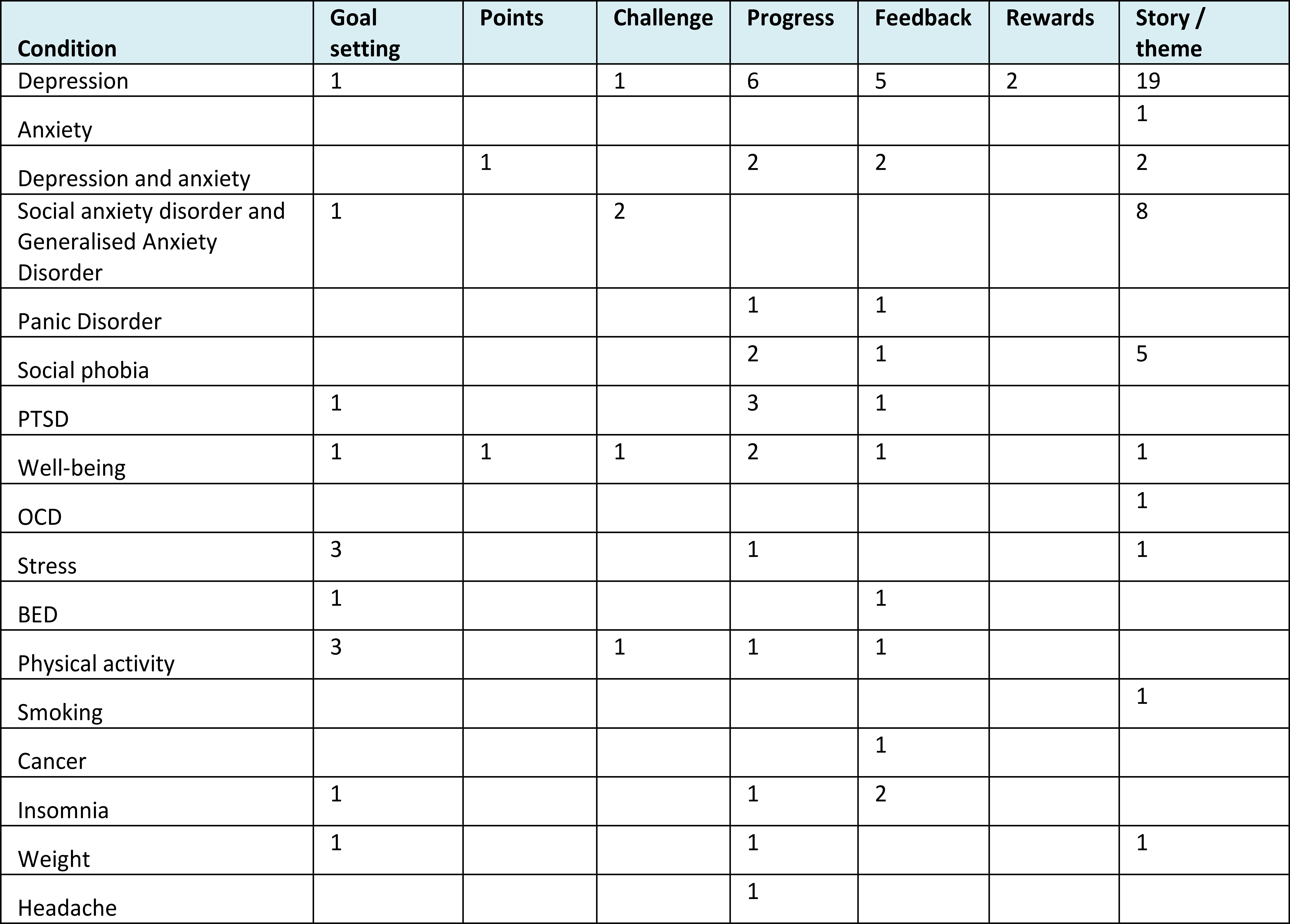

Supplement: Multimedia Appendix 6 [file mental_v3i3e39_app6.png]

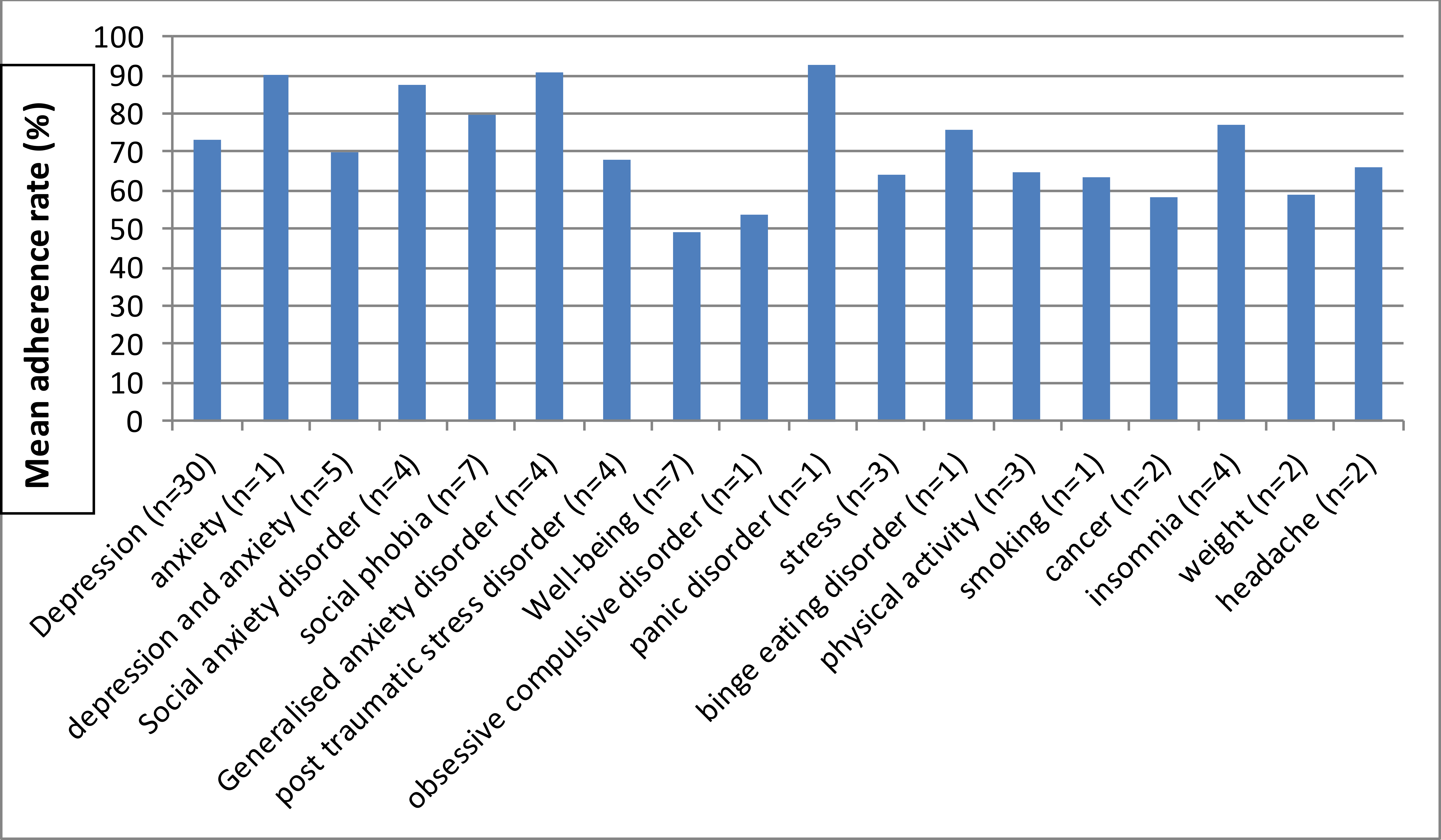

Supplement: Multimedia Appendix 7 [file mental_v3i3e39_app7.png]
