# Supplementary material for: Gamification and Adherence to Web-Based Mental Health Interventions: A Systematic Review
Source: JMIR Ment Health. 2016 Aug 24;3(3):e39. doi: 10.2196/mental.5710 (PMC5014987; doi:10.2196/mental.5710)
Supplement: Multimedia Appendix 4 [file mental_v3i3e39_app4.pdf]

Authors / year	Trial arms	Primary Condition	Active Comparator	Inactive comparator	N total	1 Gamification feature	2 Gamification features	3 Gamification feature	Automated / Guided	sequential / free navigation	Duration	Modules	Intervention Adherence to protocol %	Control Adherence to protocol %	Intervention Completion rate %
Saunders et al. 2014 [1]	2	Wellbeing	Paper based Intervention		193	Goal Setting			Automated	Sequential	>1	8	91.75	92.70	*
Williams et al. 2013 [2]	2	Depression		WLC	69	story / theme			Automated	Sequential	11	13	52.63	70.90	*
Dear, Zou et al. 2015 [3]	2	Anxiety		WLC	72	story / theme			Guided	Sequential	8	5	90	86	*
Litz et al. 2007 [4]	2	PTSD	CBT Intervention		45	Progress			Guided	Free navigation	8	7	70	n/a	*
Litz et al. 2007		PTSD	Supportive counselling Intervention			Progress			Guided	Free navigation	8	7	70	n/a	*
Knaevlsrud et al. 2007 [5]	2	PTSD		WLC	96	Goal Setting			Guided	Sequential	5	10	83.60	97.87	*
Twomey et al. 2014 [6]	2	Wellbeing		WLC	201	story / theme			Automated	Sequential	5	5	27.70	38	27.30
Anderssen, Ludstrom, Strom 2003 [7]	2	Headache	Guided plus telephone vs guided plus email		44	Progress			Guided	Free navigation	6	6	69.2	n/a	*
Anderssen, Ludstrom, Strom 2003 [7]		Headache	Guided plus telephone vs guided plus email			Progress			Guided	Free navigation	6	6	62.50	n/a	*
Andrews, Davies, Titov 2011 [8]	2	Social phobia	F-2-F		37	story / theme			Automated	Sequential	6	6	60.80	78.50	*
Carrad et al. 2011 [9]	2	BED		WLC	74	Feedback			Guided	Sequential	20	11	75.70	*	37.80
Newby et al. 2014 [10]	2	GAD		WLC	99	story / theme			Guided	Sequential	10	6	89	*	89
Powell et al. 2012 [11]	2	Wellbeing		WLC	3070	Progress			automated	Sequential	6	5	26.50	79.20	10
Imamura et al. 2014 [12]	2	Depression	Attention control (email messages)		762	story / theme			Guided	Sequential	10	6	70.90	88.20	*
Strom et al. 2013 [13]	2	Depression		WLC	48	Goal setting			Guided	Sequential	9	9	100	100	58
Beauchamp et al. 2005 [14]	2	Depression		TAU	329	Rewards			Automated	Free navigation	4	3	90.80	90.80	*
Glozier et al. 2013 [15]	2	Depression	Attention control: Health watch		562	Progress			automated	Sequential	12	12	93	99	*
Titov, Andrews, Choi, Schwencke, Johnston 2009 [16]	2	SAD	Automated		168	story / theme			Automated	Sequential	8	6	89.20	n/a	66
Titov, Andrews, Choi, Schwencke, Johnston 2009 [16]		SAD	Auto plus telephone reminders			story / theme			Automated	Sequential	8	6	85.70	n/a	78

* Not reported

References

1. Sanders M, Baker S, and Turner K. A randomized controlled trial evaluating the efficacy of Triple P Online with parents of children with early-onset conduct problems. Behaviour research and therapy 2012 50:675-84 doi: 10.1016/j.brat.2012.07.004.
2. Williams AD, Blackwell S, Mackenzie A, and Holmes E. Combining imagination and reason in the treatment of depression: a randomized controlled trial of internet-based cognitive-bias modification and internet-CBT for depression. Journal of Consulting and Clinical Psychology 2013 81(5):793-799. PMID:23750459
3. Dear B, Zou J, Ali S, lorian C, Johnston J, Sheehan J, Staples L, Gandy M, and Fogliati V. Clinical and cost effectiveness of therapist guided internet delivered cognitive behaviour therapy for older adults with symptoms of anxiety: A randomised controlled trial. Behaviour therapy 2015 46:206-217 PMID:25645169
4. Litz B, Engel CC, Nryant RA, and Papa A. A randomized, controlled proof-of-concept trial of an Internet-based, therapist-assisted self-management treatment for posttraumatic stress disorder. American journal of psychiatry 2007 164:1676-83 PMID:17974932
5. Knaevelsrud C, and Maercker A. Internet-based treatment for PTSD reduces distress and facilitates the development of a strong therapeutic alliance: a randomized controlled clinical trial. BMC Psychiatry 2007 7:13 PMID:17442125
6. Twomey C, Reilly G, Byrne M, Bury M, White A, Kissane S, McMahon A, and Clancy N. A randomized controlled trial of the computerized CBT programme, MoodGYM, for public mental health service users waiting for interventions. British Journal of Clinical Psychology 2014 53(4):433-450 PMID:24831119
7. Andersson G, Ludstrom P, and Strom L. Internet based treatment of headache: Does telephone contact add anything? Headache 2003 43:353-361 PMID:12656706
8. Andrews G, Davies M, and Titov N. Effectiveness randomized controlled trial of face to face versus Internet cognitive behaviour therapy for social phobia. Australian and New Zealand Journal of Psychiatry 2011 45:337-40 PMID:21323490
9. Carrard I, Crepin C, Rouget P, Lam T, Golay A, and Van der Linden M. Randomised controlled trial of a guided self-help treatment on the internet for binge eating disorder. Behaviour Research and Therapy 2011 49:482-91 PMID:21641580
10. Newby J, Williams A, and Andrews G. Reductions in negative repetitive thinking and metacognitive beliefs during transdiagnostic internet cognitive behavioural therapy (iCBT) for mixed anxiety and depression. Behaviour Research and Therapy 2014 59:52-60 PMID:24997439
11. Powell J, Hamborg T, Stallard N, Burls A, McSorley J, Bennett K, Griffiths KM, and Christenssen H. Effectiveness of a web-based cognitive-behavioral tool to improve mental well-being in the general population: randomized controlled trial. Journal of Medical Internet Research 2013 15:e2 PMID:23302475
12. Imamura K, Kawakami N, Furukawa T, Matsuyama Y, Shimazu A, Umandan R, Kawakami S, and Kasai K. Effects of an internet-based cognitive behavioral therapy (iCBT) program in Manga format on improving subthreshold depressive symptoms among healthy workers: a randomized controlled trial. Plos One 2014 9(5):e97167-e97167. PMID:24844530
13. Strom M, Uckelstam C, Andersson G, Hassmen P, Umefjord G, and Carlbring P. Internet-delivered therapist-guided physical activity for mild to moderate depression: A randomized controlled trial. PeerJ 2013 PMID:24109561
14. Beauchamp N, Irvine B, Seeley J, and Johnson B. Worksite-based internet multimedia program for family caregivers of persons with dementia. Gerontologist 2005 45:793-801. PMID:16326661
15. Glozier N, Christenssen H, Naismith S, Cockayne N, Donkin L, NealB, Mackinnon A, and Hickie I. Internet-Delivered Cognitive Behavioural Therapy for Adults with Mild to Moderate Depression and High Cardiovascular Disease Risks: A Randomised Attention-Controlled Trial. PloS one 2013 8 PMID:23555624

16. Titov N, Andrews G, Choi I, Schwencke G, and Johnston L. Randomized controlled trial of web-based treatment of social phobia without clinician guidance. Australian and New Zealand Journal of Psychiatry 2009 43(10):913-919. doi: 10.1080/00048670903179160
17. Titov N, Andrews G, Johnston L, Robinson E, and Spence J. Transdiagnostic internet treatment for anxiety disorders: A randomized controlled trial. Behaviour Research and Therapy 2010 48:890-9 doi: 10.1016/j.brat.2010.05.014.
18. Lintvedt O, Griffiths KM, Sorensen K, Ostvik AR, Wang CEA, Eisemann M, and Waterloo K. Evaluating the effectiveness and efficacy of unguided internet-based self-help intervention for the prevention of depression: a randomized controlled trial. Clinical Psychology & Psychotherapy 2013 20:10-27 PMID:21887811
19. Hoifodt R, Lillevoll KR, Griffiths KM, Wilsgaard T, Eismann M, Waterloo K, and Kolstrup N. The clinical effectiveness of web-based cognitive behavioral therapy with face-to-face therapist support for depressed primary care patients: randomized controlled trial. Journal of Medical Internet Research 2013 15:e153 PMID:23916965
20. Watts S, Mackenzie A, Thomas C, Griskaitis A, Mewton L, Williams A, and Andrews G. CBT for depression: a pilot RCT comparing mobile phone vs. computer. BMC Psychiatry 2013 13:49 PMID:23391304
21. Berger T, Hohl E, and Caspar F. Internet-based treatment for social phobia: a randomized controlled trial. Journal of Clinical Psychology 2009 65:1021-35 doi: 10.1002/jclp.20603. PMID:19437505
22. Mahoney A, Mackenzie A, Williams A, Smith J, and Andrews G. Internet cognitive behavioural treatment for obsessive compulsive disorder: A randomised controlled trial. Behaviour research and therapy 2014 63:99-106 PMID:25461784
23. Herbec A, Brown J, Tombor I, Michie S, and West R. Pilot randomized controlled trial of an internet-based smoking cessation intervention for pregnant smokers ('MumsQuit'). Drug and Alcohol Dependence 2014 140:130-6 PMID:24811202
24. 34. Mananes G, and Vallejo M. Usage and effectiveness of a fully automated, open-access, Spanish Web-based smoking cessation program: randomized controlled trial. Journal of Medical Internet Research 2014 16:e111 PMID:24760951
25. 35. Antypas K, and Wangberg S. E-Rehabilitation - an internet and mobile phone based tailored intervention to enhance self-management of cardiovascular disease: study protocol for a randomized controlled trial. BMC Cardiovascular Disorders 2012 12:50 PMID:22776554
26. Zetterqvist K, Maanmies J, Strom L, and Andersson G. Randomized controlled trial of internet-based stress management. Cognitive Behaviour Therapy 2003 32:151-60 PMID:16291546
27. Schover LR, Yuan Y, Fellman BM, Odensky E, Lewis PE, and Martinetti P. Efficacy trial of an internet-based intervention for cancer-related female sexual dysfunction. JNCCN Journal of the National Comprehensive Cancer Network 2013 11:1389-1397 PMID:24225972
28. Ritterband LM, Bailey ET, Thorndike FP, Lord HR, Farrell LV, and Baum LD. Initial evaluation of an Internet intervention to improve the sleep of cancer survivors with insomnia. Psycho-oncology, 2012. 21:695-705 PMID:21538678
29. Titov N, Andrews G, Schwencke G, Solley K, Johnston L, and Robinson E. An RCT comparing effect of two types of support on severity of symptoms for people completing internet-based cognitive behaviour therapy for social phobia. Australian and New Zealand Journal of Psychiatry, 2009. 43(10):920-926. PMCID: PMC2882336
30. Greene GW, White AA, Hoerr SL, Lohse B, Schembre SM, Riebe D, Patterson J, Cattleman KK, Shoff S, Horacek T, Blissmer B, and Phillips BW. Impact of an online healthful eating and physical activity program for college students. American Journal of Health Promotion: AJHP 2012 27(2):e47-e58. PMID:23113786
31. Kajiyamaa B, Thompson LW, Eto-Iwasea T, Yamashitaa M, Di Marioa J, Tzuangc YM, and Gallagher-Thompson D. Exploring the effectiveness of an Internet-based program for reducing caregiver distress using the iCare Stress Management e-Training Program. Aging and mental health 2013 17(5) PMID:23461355
32. Cooper CL, Hind D, Parry GD, Isaac C, Dimairo M, O'Cathain A, Rose A, Freeman JV, Martin L, Kaltenthaler EC, Thake A, and Sharrack B. Computerised cognitive behavioural therapy for the treatment of depression in people with multiple sclerosis: external pilot trial. Trials 2011 12:259-259 PMID:22168507
33. Brief D, Rubin A, Keane TM, Enggasser JL, Roy M, Helmuth E, Hermos J, Lachowicz M, Rybin D, and Rosenbloom D. Web intervention for OEF/OIF veterans with problem drinking and PTSD symptoms: A randomized clinical trial. Journal of Consulting and Clinical Psychology 2013 81:890-900 PMID:23875821

34. Richards D, Timulak L, and Hevey D. A comparison of two online cognitive-behavioural interventions for symptoms of depression in a student population: The role of therapist responsiveness. Counselling & Psychotherapy Research 2013 13(3):184-193. doi:10.1080/14733145.2012.733715
35. Thorndike F, Ritterband LM, Gonder-Frederick LA, Lord HR, Ingersoll KS, and Morin CM. A randomized controlled trial of an internet intervention for adults with insomnia: effects on comorbid psychological and fatigue symptoms. Journal of Clinical Psychology 2013 69:1078-93 PMID:24014057
36. Aikens KA, Pelletier K, and Baase C. Mindfulness goes to work: Impact of an online workplace intervention. Journal of Occupational and Environmental Medicine 2014 56(7):721-731 PMID:24988100
37. Santucci LC, McHugh RK, Elkins RM, Schechter B, Ross MS, Landa CE, Eisen SE, and Barlow DH. Pilot implementation of computerized cognitive behavioral therapy in a university health setting. Administration and Policy in Mental Health and Mental Health Services Research 2014 41(4):514-521. PMID:23592231
38. Carlbring P, Bohman S, Brunt S, Burhman M, Westling BE, Ekselius L, and Andersson G. Remote treatment of panic disorder: a randomized trial of internet-based cognitive behavior therapy supplemented with telephone calls. American Journal of Psychiatry 2006 163:2119-25 PMID:17151163
39. Moritz S, Schilling L, Hauschildt M, Schroder J, and Treszl A. A randomized controlled trial of internet-based therapy in depression. Behaviour research and therapy 2012 50:513-21 PMID:22677231
40. Bossen D, Veenhof C, Van Beek KEC, Spreeuwenberg PMM, Dekker J, and De Bakker DH. Effectiveness of a web-based physical activity intervention in patients with knee and/or hip osteoarthritis: randomized controlled trial. Journal of Medical Internet Research 2013 15(11):e257-e257. PMID:24269911
41. Cook R, Billings DW, Hersch RK, Back AS, and Hendrickson A. A field test of a web-based workplace health promotion program to improve dietary practices, reduce stress, and increase physical activity: randomized controlled trial. Journal of Medical Internet Research 2007 9(2):e17. PMID:17581811
42. Phillips R, Schneider J, Molosankwe I, Leese M, Foroushani PS, Grime P, McCrone P, Morriss R, and Thornicroft G. Randomized controlled trial of computerized cognitive behavioural therapy for depressive symptoms: effectiveness and costs of a workplace intervention. Psychological Medicine 2014 44(4):741-752. doi: 10.1017/S0033291713001323
43. Irvine A, Gelatt V, Seeley J, Macfarlane P, Gau J. Web-based intervention to promote physical activity by sedentary older adults: randomized controlled trial. Journal of Medical Internet Research 2013 15:e19 PMID:23470322
44. Sheeber L, Seeley JR, Feil EG, Davis B, Sorensen E, Kosty DB, and Lewinsohn PM. Development and pilot evaluation of an internet-facilitated cognitive-behavioral intervention for maternal depression. Journal of Consulting and Clinical Psychology 2012 80:739-49 PMID:22663903
45. Cohn MA, Pietrucha ME, Saslow LR, Hult JR, and Moskowitz JT. An online positive affect skills intervention reduces depression in adults with type 2 diabetes. Journal of Positive Psychology 2014 9(6):523-534. PMID: 25214877
46. Cobb N, and Poirier J. Effectiveness of a multimodal online well-being intervention: A randomized controlled trial. American journal of preventive medicine 2014 46:41-8 PMID:24355670
47. Titov N, Andrews G, Schwencke G, Robinson E, Peters L, and Spence J. Randomized controlled trial of Internet cognitive behavioural treatment for social phobia with and without motivational enhancement strategies. Australian and New Zealand Journal of Psychiatry 2010 44:938-45 PMID:20932208
48. Mackinnon A, Griffiths K, and Christensen H. Comparative randomised trial of online cognitive-behavioural therapy and an information website for depression: 12-month outcomes. British Journal of Psychiatry 2008 192:130-4 PMID:18245031
49. Robinson E, Titov N, Andrews G, McIntyre K, ASchwencke G, and Solley K. Internet treatment for generalized anxiety disorder: a randomized controlled trial comparing clinician vs. technician assistance. PloS one 2010 5:e10942 PMID:20532167
50. Clarke G, Eubanks D, Kelleher C, O'Connor E, DeBar LL, Lynch F, Nunley S, and Guillion C. Overcoming Depression on the Internet (ODIN) (2): A randomized trial of a self-help depression skills program with reminders. Journal of Medical Internet research 2005 7, e16 PMID:15998607

51. Mitchell J, Stanimirovic R, Klein B, and Vella-Brodrick D. A randomised controlled trial of a self-guided internet intervention promoting well-being. Computers in Human Behavior 2009 25(3):749-760 PMID:24132044
52. Proudfoot J, Clarke J, Birch M, Whitton AE, Parker G, Manicavasgar V, Harrison V, Christensen H, and Hadzi-Pavlovic D. Impact of a mobile phone and web program on symptom and functional outcomes for people with mild-to-moderate depression, anxiety and stress: a randomised controlled trial. BMC Psychiatry 2013 13:312 PMID:24237617
53. Thompson N, Walker ER, Obolensky N, Winning A, Barmon C, Dilorio C, and Compton M. Distance delivery of mindfulness-based cognitive therapy for depression: project UPLIFT. Epilepsy & Behavior 2010 19:247-54 PMID:20851055
54. Titov N, Dear BF, Johnston L, Lorian C, Zou J, Wootton B, Spence J, McEvoy PM, and Rapee RM. Improving Adherence and Clinical Outcomes in Self-Guided Internet Treatment for Anxiety and Depression: Randomised Controlled Trial. PloS one 2013 8(7). PMID:23843932
55. Titov N, Andrews G, Davies M, McIntyre K, Robinson E, and Solley K. Internet treatment for depression: a randomized controlled trial comparing clinician vs. technician assistance. PloS one 2010 5:e10939 doi: 10.1371/journal.pone.0010939.
56. Berger T, Hammerli K, Gubser N, Gerhard A, and Caspar F. Internet-based treatment of social phobia: a randomized controlled trial comparing unguided with two types of guided self-help. Behaviour Research and Therapy 2011 49:158-69 PMID:21255767
57. Titov N, Andrews G, Choi I, Schwencke G, and Mahoney A. Shyness 3: randomized controlled trial of guided versus unguided Internet-based CBT for social phobia. Australian and New Zealand Journal of Psychiatry 2008 42:1030-40 doi: 10.1080/00048670802512107.
58. Botella C, Gallego MJ, Garcia-Palacios A, Guillien V, Banos RM, Quero S, and Alcaniz M. An Internet-based self-help treatment for fear of public speaking: a controlled trial. Cyberpsychology, behavior and social networking 2010 13:407-21 PMID:20712499
59. Ho FY-Y, Chung K-F, Yeung W-F, Ng T H-Y, and Cheng S K-W. Weekly brief phone support in self-help cognitive behavioral therapy for insomnia disorder: Relevance to adherence and efficacy. Behaviour Research and Therapy 2014 63:147-156 PMID:25461790
60. Farrer L, Christensen H, Griffiths KM, and Mackinnon A. Internet-based CBT for depression with and without telephone tracking in a national helpline: randomised controlled trial. PloS one, 2011. 6:e28099 PMID:22140514
61. Christensen H, Griffiths KM, Mackinnon AJ, and Brittliffe K. Online randomized controlled trial of brief and full cognitive behaviour therapy for depression. Psychological Medicine 2006. 36(12):1737-1746. PMID:16938144
